# Functional correlates of TSH, fT3 and fT4 in Alzheimer disease: a F-18 FDG PET/CT study

**DOI:** 10.1038/s41598-017-06138-7

**Published:** 2017-07-24

**Authors:** Agostino Chiaravalloti, Francesco Ursini, Alessandro Fiorentini, Gaetano Barbagallo, Alessandro Martorana, Giacomo Koch, Mario Tavolozza, Orazio Schillaci

**Affiliations:** 1 Department of Biomedicine and Prevention, University Tor Vergata, Rome, Italy; 2 IRCCS Neuromed, Pozzilli (IS), Italy; 3 Department of Health Sciences, University Magna Graecia, Catanzaro, Italy; 4 Institute of Neurology, University Magna Graecia, Catanzaro, Italy; 5 Department of Neurosciences, University Tor Vergata, Rome, Italy; 6 IRCCS Santa Lucia, Rome, Italy

## Abstract

The present study was aimed to investigate the relationships between thyroid stimulating hormone (TSH), freeT3 (fT3) and freeT4 (fT4) and brain glucose consumption as detectable by means of 2-deoxy-2-(F-18) fluoro-D-glucose (F-18 FDG) Positron Emission Tomography/Computed Tomography (PET/CT) in a selected population with Alzheimer disease (AD). We evaluated 87 subjects (37 males and 50 females, mean age 70 (±6) years old) with AD. All of them were subjected to TSH, fT3 and fT4 assay and to cerebrospinal fluid amyloid (Aβ1-42) and tau [phosphorylated-tau (p-tau) and total-tau (t-tau)] assay prior PET/CT examination. Values for TSH, fT3 and fT4 were in the normal range. The relationships were evaluated by means of statistical parametric mapping (SPM8) using age, sex, MMSE, scholarship and CSF values of amyloid and tau as covariates. We found a significant positive correlation between TSH values and cortical glucose consumption in a wide portion of the anterior cingulate cortex bilaterally (BA32) and left frontal lobe (BA25) (p FWE-corr <0.001; p FDRcorr <0.000; cluster extent 66950). No significant relationships were found between cortical F-18 FDG uptake and T3 and T4 serum levels. The results of our study suggest that a cortical dysfunction in anterior cingulate and frontal lobes may affect serum values of TSH in AD patients.

## Introduction

Alzheimer’s disease (AD) is a neurodegenerative disorder leading to cognitive decline and dementia. The pathogenic mechanisms are still under study. However, the leading hypothesis postulates that metabolic change of a membrane protein, the amyloid precursor protein, could be reasonably responsible for most of the pathological changes observed in course of AD (amyloid hypothesis)^1^. Paired-helical filaments of hyper-phosphorylated Tau compose the neuro-fibrillary tangles^2, 3^. These pathological structures are usually located inside dendrites and linked from the neuropil through dendritic shafts to the neuronal cell bodies which contain the neuro-fibrillary tangles^4^. It is suggested that the pathological aggregation of these proteins is related to neuronal toxicity and cell loss, since Tau aggregate spreading in AD brain increases with cognitive decline^5^.

In the functioning of human brain, normal thyroid status is essential for the maintenance of optimal cognitive performance^6^. The “classical” concept of an inverse relation between thyroid function and cognitive abilities has been claimed for decades^7^ and an inverse correlation between thyroid stimulating hormone (TSH) and mild cognitive impairment (MCI) has been inconsistently demonstrated^8, 9^. However, novel evidence reappraised this milestone concept and large, well-designed studies have voided the theory that subtle thyroid abnormalities, such as subclinical hypothyroidism, could cause cognitive impairment^10^. From the other side, some evidence points to a beneficial effect of L-thyroxine supplementation in cognitive function^11^.

To complicate matters, the link between AD and thyroid function is a much more complicated story. Early evidences suggested a perturbation of thyrotropin-releasing hormone (TRH)-TSH axis, characterized by a blunted response of TSH levels after stimulation with exogenous TRH^12–14^. However, a number of other studies failed to confirm this association^15, 16^. More recently, an epidemiological link between thyroid function and AD development was emphasized from different groups. For example, in the Rotterdam study, a large population-based longitudinal study, patients with low baseline TSH levels had higher risk of dementia and AD^17^. Also in patients with MCI, low TSH correlated with the risk of developing AD in a 6-years follow-up study^18^. Similar findings were confirmed in different populations^19^. In addition to baseline levels, also the normal circadian rhythm of TSH release seems to be disrupted in AD^20^.

The usefulness of 2-deoxy-2-(^F-18^) fluoro-D-glucose (F-18 FDG) Positron Emission Tomography/Computed Tomography (PET/CT) in the diagnosis of AD an in its differential diagnosis from other neurodegenerative diseases has been widely investigated showing a good sensitivity, specificity, and diagnostic accuracy in the detection of bilateral temporo-parietal hypometabolism associated with AD^21–23^.

Aim of this study was to investigate the relationships among TSH, fT3 and fT4 and brain glucose consumption as detectable by means of F-18 FDG PET/CT in a population with clinical diagnosed AD.

## Materials and Methods

Informed consent was obtained from all of the patients and procedures followed were in accordance with the ethical standards of the responsible committee on human experimentation (institutional and national) and with the Helsinki Declaration of 1975, as revised in 2008^24^. The Ethics Committee of the Policlinico Tor Vergata approved the protocol research.

### AD Patients

For the present study, we examined 87 newly clinical-diagnosed AD patients according to the NINCDS-ADRDA criteria^25^. An outline of the patients examined is provided in Table 1. All patients underwent a complete clinical investigation, including medical history and neurological and neuropsychological examination, laboratory evaluation^26^. Structural Magnetic Resonance (MR) was performed within 1 month prior F-18-FDG PET/CT brain scan in order to exclude any possible brain lesion and to aid in image analysis (when required, PET and MR data were co-registered in order to exclude any artifact, partial volume effect as in the case of an expanded cerebral sulcus^27^). Exclusion criteria were the following: 1) patients with isolated deficits and/or unmodified MMSE (≥25/30) on revisit (6, 12 and 18 months follow-up); 2) patients with clinically manifest acute stroke in the last 6 months showing an Hachinsky scale >4 and a radiological evidence of sub-cortical lesions; 3) patients with known history of hypothalamus disease, appendices suprasphenoidalis disease, or thyroid disease or taking L-tiroxine substitution therapy or anti-thyroid therapy. None of patients revealed pyramidal and/or extrapyramidal signs at the neurological examination. Moreover, patients with a past history of diabetes, cancer, HIV or with previous brain surgery, radiation or trauma were excluded from the study. Accordingly 102 patients were evaluated and 15 were excluded because presented one or more exclusion criteria or refused to give informed consent.

**Table 1:** General overview of the AD population examined, including sociodemographic variables.

	AD (n = 87)	CG (n = 13)	*p*-value
Age	70 ± 6	71 ± 6	0.28
Male sex	37	41	ns
Education: BUL	68	59	ns
Education: ULoA	19	21	
Occupation: M	70	65	ns
Occupation: S	17	15	
MMSE	18.9 ± 7.2	28.4 ± 1.5	<0.0001
Aβ_1-42_ (pg/ml)	344.28 ± 134.31	818 ± 202.7	<0.0001
p-Tau (pg/ml)	96.7 ± 76.9	40.3 ± 10.9	<0.0001
t-Tau (pg/ml)	679.15 ± 330.5	272 ± 84.2	<0.0001
TSH (uU/ml)	1.38 ± 0.84	1.58 ± 0.73	0.10
FT3 (pg/ml)	3.04 ± 0.33	3.18 ± 0.27	0.004
FT4 (ng/ml)	1.16 ± 0.17	1.22 ± 0.21	0.08

BUL: below university level; ULoA: university level or above; M: manual; S: skilled.

All AD patients showed a cognitive profile consistent with dementia, as assessed by a neuropsychological evaluation including the MMSE and a standardized neuropsychological battery (see below).

We did not consider patients undergoing treatment with drugs that could interfere with F-18-FDG uptake and distribution in the brain and, in particular, all AD patients were discontinued cholinesterase inhibitor treatment throughout the study^28^.

### Control group

For comparison, we enrolled a group of 80 age- and sex- matched euthyroid control subjects (CG) aged >65 years in whom AD was clinically excluded by extensive evaluation from an experienced neurologist (A.M.). All the CG patients underwent TSH, fT3 and fT4 measurements. Of them, only thirteen underwent CSF collection and analysis and aF-18 FDG PET/CT scan (see Table 1). In particular all of them were chemotherapy naïve. Before their inclusion in our study, all control subjects underwent MRI evaluation, performed 7 ± 2 days before PET/CT examination, in order to exclude brain injury.

### Cognitive Evaluation

At the time of enrollment, all recruited patients underwent a neuropsychological battery including the following cognitive domains: general cognitive efficiency: MMSE^29^; verbal episodic long-term memory: Rey auditory verbal long term memory (RAVLT) (15-Word List Immediate and 15-min Delayed recall)^30^; visuo-spatial abilities and visuo-spatial episodic long-term memory: Complex Rey’s Figure (copy and 10-min Delayed recall)^31^; executive functions: phonological word fluency (PWF)^32^; analogic reasoning: Raven’s Colored Progressive Matrices (RCPM)^33^. For all employed tests, we used the Italian normative data for both score adjustment (gender, age, and education) and to define cut-off scores of normality, determined as the lower limit of the 95% tolerance interval. For each test, normative data are reported in the corresponding references.

### CSF collection and analysis

All the CSF samples were obtained by lumbar puncture (LP) performed at rest, in decubit-position, between 9:00 and 10:00 AM, after overnight fasting, using an atraumatic needle. Blood specimens were also obtained at the same time of LP procedure. CSF samples were collected in polypropylene tubes using standard sterile techniques. The first 4 ml CSF sample was used for biochemistry routine analysis including total cell count and lactate levels. A second 4 ml CSF sample was centrifuged to eliminate cells and cellular debris and immediately frozen at −80 °C until the analysis to assess t-Tau, p-Tau and Aβ_1-42_ amounts, performed as previously described. Chemistry assays were carried out using commercially available kits following the manufacturer’s specifications (Innotest β-Amyloid 1–42, Innotest h-T-tau, InnotestPhospho-T-tau 181; Innogenetics, Ghent, Belgium).

### Laboratory evaluation

Venous blood (5 mL) was collected in fasting state. Serum was prepared within 60 min of blood collection. Serum was store at −20 °C until analyzed for thyroid function test.

These samples were analyzed within 24 h from blood collection time. Thyroid stimulating hormone (TSH), serum free 3,5,3′-triiodothyronine (fT3) and free 3,5,3′,5′-tetraiodothyronine (fT4) were analyzed. Measurements of fT3 and fT4 were based on a direct, labeled antibody, competitive immunoassay, while TSH assay was based on one-step immunoenzymatic sandwich principle in conjunction with biotin-streptavidin technology.

### F-18 FDG injection and PET/CT scan

The PET/CT system Discovery VCT (GE Medical Systems, Tennessee, USA) has been used to assess F-18 FDG brain distribution in all patients by means of a 3D-mode standard technique in a 256 × 256 matrix. Reconstruction was performed using the 3-dimensional reconstruction method of ordered-subsets expectation maximization (OSEM) with 20 subsets and with 4 iterations. The system combines a high-speed ultra 16-detector-row (912detectors per row) CT unit and a PET scanner with 13440 bismuth germanate crystals in 24 rings (axial full width at half-maximum 1-cm radius, 5.2 mm in 3D mode, axial field of view 157 mm). A low-ampere CT scan of the head for attenuation correction (40 mA; 120 Kv) was performed before PET image acquisition. All the subjects fasted for at least 5 h before intravenous injection of F-18 FDG; the serum glucose level was up to 95 mg/ml in all of them. All the subjects were injected intravenously with 185–210 MegaBequerels of F-18 FDG and hydrated with 500 ml of saline (0.9% sodium chloride). PET/CT acquisition was started 30 minutes after F-18 FDG injection^34^.

### Statistical analysis

Correlations among brain F-18 FDG uptake, clinical and CSF data were analyzed using statistical parametric mapping (SPM8, Wellcome Department of Cognitive Neurology, London, UK) implemented in Matlab R2012b (Mathworks, Natick, Massachusset, USA). MMSE scores, sex, age and CSF biomarkers were used as covariates in each correlation analysis. F-18 FDG PET data have been subjected to affine and non-linear spatial normalization into the Montreal Neurological Institute space. An aging and dementia-specific template for spatial normalization was used^35^. The spatially normalized set of images were then smoothed with a 8 mm isotropic Gaussian filter to blur for individual variations in gyral anatomy and to increase the signal-to-noise ratio. Images have been globally normalized to 50 using proportional scaling to remove confounding effects to global cerebral glucose consumption changes, with a masking threshold of 0.8. The resulting statistical parametric maps, SPM[t], have been transformed into normal distribution (SPM[z]) unit. Correction of SPM coordinates to match the Talairach coordinates was achieved by the subroutine implemented by Matthew Brett (http://www.mrc-cbu.cam.ac.uk/Imaging). Brodmann areas (BA) have been identified at a range from 0 to 3 mm from the corrected Talairach coordinates of the SPM output isocentres, after importing the corrected coordinates, by Talairach client (http://www.talairach.org/index.html). According to Bennett et collegues^36^, SPM t-maps have been set at p < 0.05, corrected for multiple comparisons with the False Discovery Rate option at voxel level, and at p < 0.01 corrected for multiple comparison at cluster level. Only those clusters containing more than 100 (5 × 5 × 5 voxels, i.e. 11 × 11 × 11 mm) contiguous voxels have been accepted as significant. For correlation analyses in both AD and CG subjects, the voxel-based analyses have been performed using a ‘regression analysis’ design model using sex, age, MMSE and CSF parameters presented in Table 1 as covariates. In SPM maps, we searched the brain areas with a significant correlation using a statistical threshold of p = 0.001, family wise error-corrected for the problem of multiple comparisons, with an extent threshold of 100 voxels.

The cluster obtained by this comparison has been exported and further analyzed after a normalization process. In particular, the mean signal intensities computed of each cluster have been normalized within each subject to the average intensities of the cerebellar Volume of Interest as defined by Schmahmann *et al*.^37^. For this purpose, WFU_Pickatlas was used^38, 39^. This choice was based on the knowledge that AD pathological processes poorly affect the cerebellum and on the evidence that, when using cerebellum instead of whole brain counts as the reference region, accuracy in distinguishing AD patients from controls increases^40^. As proposed in another report of Pagani *et al*.^41^, a dataset including cerebellum-normalized F-18 FDG PET values relevant to the examined cluster has been exported. In order to assess that cerebellum-normalized F-18 FDG PET values for the cluster examined were of Gaussian distribution, D’Agostino K squared normality test has been applied (where the null hypothesis is that the data are normally distributed). Spearman’s correlation and linear regression were used in order to investigate the relationships among cerebellum-normalized F-18 FDG PET values, thyroid hormones (TSH, T3, T4) and CSF parameters. An hypothesis was considered valid when P-Value was less or equal than 0.05.

Differences in CSF parameters, thyroid hormone values and socio demographic variables in sex were evaluated by means of Mann Whitney U test or Two way ANOVA when appropriate (see Table 1).

A ‘two-sample t test’ design model was used for the following voxel-based comparisons between AD and CG: AD versus CG and vice versa.

### Connectivity analysis

Since according to Horowitz *et al*. all brain regions whose glucose metabolism is correlated at rest are functionally associated^42^, a connectivity correlation analysis was performed based on voxel-wise interregional correlation analysis (IRCA) of SPM using resting F-18 FDG PET data using population-based cytoarchitectonic seed brain regions as proposed previously^43^. In particular, for IRCA, extracted mean regional VOI counts were then normalized (see above) and used as regression factor to find regions showing significant (corrected <0.05) voxel-wise correlations across scans (subjects) using SPM8^43^.

## Results

### General characteristics of AD patients and controls

General characteristics of the study population are summarized in Table 1. AD patients and CG did not differed in age, sex, and education level. AD patients showed a trend towards reduced TSH (1.38 ± 0.84 vs 1.58 ± 0.73, *p* = 0.10) and fT4 (1.17 ± 0.17 vs 1.22 ± 0.21, *p* = 0.08) levels, although not reaching the statistical significance; in contrast fT3 was significantly lower (3.04 ± 0.33 vs 3.18 ± 0.27, *p* = 0.004).

### PET/CT scan in AD and controls

When subtracting AD to CG subjects, SPM analysis showed a significant reduction of brain glucose consumption in a wide portion of right and left temporal, parietal and frontal cortex in AD patients (Table 2).

**Table 2:** Statistical parametric mapping comparisons between ^18^F-FDG uptake in CG and AD.

**Analysis**	Cluster level					Voxel level		
	cluster p (FWE-corr)	cluster p (FDR-corr)	Cluster extent	Cortical Region	Z score of maximum	Talairach coordinates	Cortical region	BA
CG - AD	0.000	0.000	17423	R Parietal	5.19	2, −48, 34	Precuneus	7
				R Parietal	5.04	52, −50, 46	Inferior parietal lobule	40
				R Temporal	5.00	62, −34, −2	Middle temporal gyrus	21
	0.000	0.000	14169	L Parietal	5.35	0, −48, 34	Precuneus	31
				L Temporal	5.17	−54, −28, −10	Middle temporal gyrus	21
				L Temporal	5.05	−52, −54, −14	Inferior temporal gyrus	20
				L Frontal	4.98	−8, 18, −14	Medial frontal gyrus	25

In the ‘cluster level’ section on left, the number of voxels, the corrected P value of significance and the cortical region where the voxel is found, are all reported for each significant cluster. In the ‘voxel level’ section, all of the coordinates of the correlation sites (with the Z-score of the maximum correlation point), the corresponding cortical region and BA are reported for each significant cluster. L, left; R, right; BA, Brodmann’s area. In the case that the maximum correlation is achieved outside the grey matter, the nearest grey matter (within a range of 5 mm) is indicated with the corresponding BA.

### Correlation analysis between TSH and clinical features

In AD patients, no significant correlation were found between TSH and age, sex, MMSE, amyloid-β 1-42, Tau, and p-Tau. In particular, no correlation was found between TSH and fT3 (R = 0.05, p = 0.61) or fT4 (R = 0.03, p = 0.81). In CG, TSH was significantly correlated with fT3 (R = −0.24, p = 0.03) and fT4 (R = −0.26, p = 0.02), but not with age.

### Correlation between TSH and PET/CT scan in AD patients

**Figure 1:**
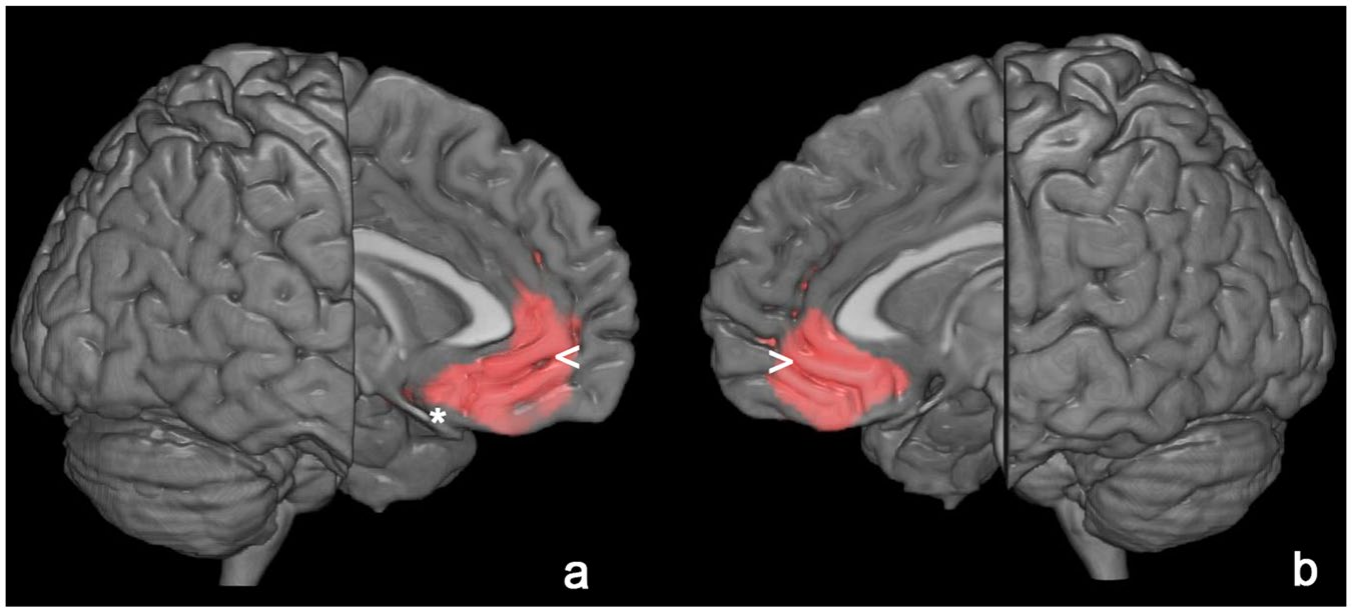
3D rendering of data presented in Table 3 in (**a**) showing the positive correlation between TSH and brain glucose consumption in left anterior cingulate cortex (<) and medial frontal gyrus (*) and in the right anterior cingulate cortex (**b**, >). Threshold P < 0.01 corrected for multiple comparisons with false discovery rate at the voxel level. Coordinate and other regional details are presented in Table 3.

In AD patients, SPM analysis showed a significant positive correlation between serum TSH values and cortical glucose consumption in a wide portion of the anterior cingulate cortex bilaterally (BA 32) and left frontal lobe (BA25) (Table 3, Fig. 1). No significant correlations were found between cortical F-18 FDG uptake and fT3 and fT4 levels.

**Table 3:** Multiple regression analysis showing the TSH related areas of increased ^18^F FDG brain uptake.

**Analysis**	Cluster level					Voxel level		
	cluster p (FWE-corr)	cluster p (FDR-corr)	Cluster extent	Cortical Region	Z score of maximum	Talairach coordinates	Cortical region	BA
Positive correlation	0.001	0.000	66950	R Limbic	4.62	14, 46, −4	Anterior Cingulate	32
				L Frontal	4.14	−8, 18, −14	Medial Frontal Gyrus	25
				L Limbic	4.05	−2, 32, −10	Anterior Cingulate	32
Negative correlation	—	—	—	—	—	—	—	—

In the ‘cluster level’ section on left, the number of voxels, the corrected P value of significance and the cortical region where the voxel is found, are all reported for each significant cluster. In the ‘voxel level’ section, all of the coordinates of the correlation sites (with the Z-score of the maximum correlation point), the corresponding cortical region and Brodmann Area are reported for each significant cluster. L, left; BA, Brodmann’s area. In the case that the maximum correlation is achieved outside the grey matter, the nearest grey matter (within a range of 5 mm) is indicated with the corresponding BA.

Cerebellum-normalized F-18 FDG PET values for left BA32 were equal to 1.10 ± 0.09 (mean ± standard deviation) with a good correlation between TSH levels and normalized F-18 FDG uptake, with high levels of TSH being related to higher levels of F-18 FDG uptake (r = 0.26 and P = 0.01). As for right BA32, values for cortical glucose consumption resulted equal to 1.13 ± 0.1, with high levels of TSH being related to higher levels of F-18 FDG uptake (r = 0.27 and P = 0.01). As for left BA25, normalized F-18 FDG PET values resulted equal to 0.96 ± 0.1, with high levels of TSH being related to higher levels of F-18 FDG uptake (r = 0.29 and P = 0.005).

### Correlation between CSF parameters and PET/CT scan in AD patients

A significant correlation was found between CSF t-Tau levels and cortical metabolism in right BA32 (r = −0.25, P = 0.019) and left BA32 (r^2^ = 0.054 and P = 0.033) while no significant correlation was found in BA25 (r = −0.11; P = 0.29). We did not find significant correlation with p-Tau and cortical activity in right BA32 (r = −0.1; P = 0.34) and left BA32 (r^2^ = 0.054; P = 0.9) and BA25 (r = −0.12; P = 0.25). Similarly, we did not find significant correlation with Aβ_1-42_ and cortical activity in right BA32 (r = −0.09; P = 0.37) and left BA32 (r^2^ = 0.009; P = 0.38) and BA25 (r = −0.15; P = 0.16).

### Correlation between TSH and CSF parameters or PET/CT scan in controls

In CG undergoing both CSF assay and PET/CT examination, (13 subjects, 7 women and 6 men) CSF values were in the normal range with t-Tau, p-Tau and Aβ_1-42_ being equal to 272 ± 84.2, 40.3 ± 10.9 and 818 ± 202.7 pg/ml respectively. In these subgroup, TSH, fT3 and fT4 were equal to 1.48 ± 0.38, 2.37 ± 1.02, 1.13 ± 0.12. No correlation was found between TSH, fT3 or fT4 and t-Tau, p-Tau or Aβ_1-42_.

For converse, SPM regression analysis showed a significant positive correlation (P FWE corr.: 0.004; P FDR corr: 0.004; cluster extent: 5129) between TSH levels and cortical metabolism in the left superior temporal gyrus (BA 39, Talairach coordinates: −44, −50, 28) and in the right precuneus (BA7, Talairach coordinates: 8, −56, 64). TSH was not negatively correlated with F-18 FDG consumption. We did not find any significant relationships with fT3 and fT4 and brain metabolism.

### Connectivity analysis

**Figure 2:**
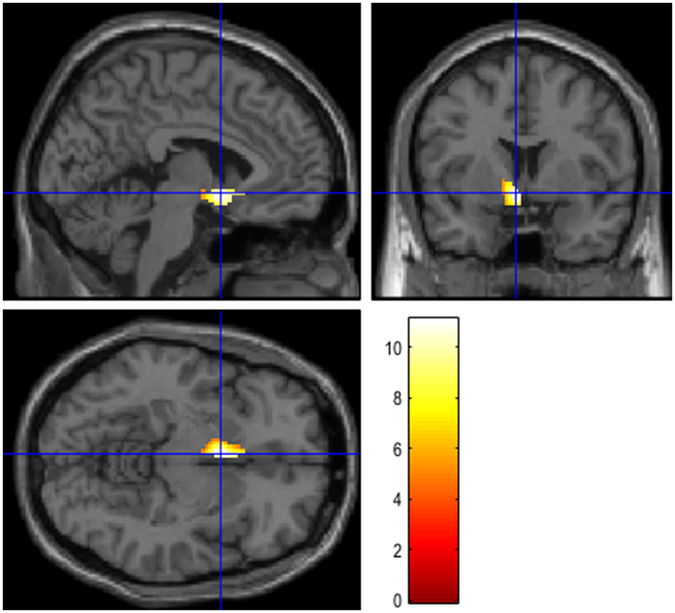
T1 magnetic resonance imaging superimposition showing the significant (positive) relationship between cortical activity in BA25 and that of the region corresponding to the hypothalamus. Since cluster was large as compared to the anatomical region here represented, for illustrative purposes a mask with WFU pickatlas was generated. Coordinates are shown in Table 4.

Connectivity analysis showed a significant *positive* correlation between glucose consumption in: a) right BA32 and a wide cortical area in frontal lobe (BA8 and 47); b) left BA25 and a left periventricular area (hypothalamus, see Fig. 2) and right temporal cortex (BA38) as shown in Table 3; c) left BA32 and a wide portion of left frontal cortex (BA6 and BA8). A significant *negative* correlation was found between: a) right BA32 and left BA13 (insula); b) BA25 and a wide portion of right parietal and frontal cortex (BA7 and BA6) and left limbic cortex (BA32); c) left BA32 and a wide portion of right frontal (BA25) and right temporal (BA21) cortex. Detailed results with Talairach coordinates are provided in Table 4.

**Table 4:** Regression analysis showing the areas of increased (positive correlation, + ) or decreased (negative correlation, −) ^18^F FDG brain uptake related to Brodmann areas 32 (right, left) and 25.

Analysis	Cluster level					Voxel level		
	cluster p (FWE-corr)	cluster p (FDR-corr)	Cluster extent	Cortical Region	Z score of maximum	Talairach coordinates	Cortical region	BA
+ BA32 (right)	0.000	0.000	32099	R Frontal	7.61	6, 30, 38	Medialfrontal gyrus	8
				R Frontal	6.11	42, 16, −8	Inferiorfrontal gyrus	47
+ BA25	0.000	0.000	10915	L Cerebrum	Inf.	−8 −6 −5	Hypothalamus	—
				R Temporal	5.44	24, 8, −24	Superior temporal gyrus	38
+ BA32 (left)				L Temporal	4.29	−44, 10, −18	Superior Temporal Gyrus	38
				L Limbic	4.22	−18, −18, −22	Parahippocampal Gyrus	28
	0.000	0.000	41654	L Frontal	Inf.	−14, 20, 58	Superior frontal gyrus	6
					7.81	−16, 14, 52	Superior frontal gyrus	6
					7.47	−18, 24, 38	Sub-gyral	8
−BA32 (right)	0.000	0.000	40269	L Cerebrum	6.94	−30, −24, 16	Insula	13
−BA25	0.000	0.000	19608	R Parietal	5.65	18, −56, 70	Postcentral gyrus	7
				R Frontal	5.45	2, −12, 66	Medialfrontal gyrus	6
				L limbic	5.41	−8, 16, 54	Cingulate gyrus	32
−BA32 (left)	0.000	0.000	16056	R Frontal	5.48	6, 12, −18	Medialfrontal gyrus	25
				R Temporal	5.05	65, −20, −4	Middle temporal gyrus	21

In the ‘cluster level’ section on left, the number of voxels, the corrected P value of significance and the cortical region where the voxel is found, are all reported for each significant cluster. In the ‘voxel level’ section, all of the coordinates of the correlation sites (with the Z-score of the maximum correlation point), the corresponding cortical region and BA are reported for each significant cluster. L, left; R, right; BA, Brodmann’s area. In the case that the maximum correlation is achieved outside the grey matter, the nearest grey matter (within a range of 5 mm) is indicated with the corresponding BA.

## Discussion

The present study examined the correlation between TSH levels within the normal range and cerebral glucose metabolism in patients with AD, showing that serum TSH levels are positively correlated with glucose uptake in bilateral anterior cingulate cortex and left medial frontal gyrus. Although literature evidence extensively explored the relationship between TSH levels and cognitive function, results are still conflicting. Previous studies^19, 44^ reported that subclinical hypothyroidism is associated with cognitive impairment, and that even individuals whose TSH levels are only marginally reduced within the normal range have an increased risk of AD. Otherwise, other investigations^45^ did not find any relationship between thyroid hormones levels and cognitive function. This discrepancy may be due to variations in the clinical characteristics of participants and the different neuropsychological measures targeting specific cognitive domains such as attention, executive function, and episodic memory. Similarly, our results showed no correlation between thyroid hormones levels and MMSE score. Hence, a more objective method than neuropsychological measures could be necessary to elucidate the influence of thyroid hormones on the pathology of AD. In this context, to objectively evaluate cerebral metabolism by using PET/TC may provide a more sensitive insight to understand the relationships between thyroid function and AD.

To the best of our knowledge, few studies have been carried out in order to investigate the relationship between thyroid hormones and brain metabolism. Interestingly, Kimura N. *et al*. found that serum levels of TSH, but not free T3 or free T4, were significantly inversely correlated with regional cerebral blood flow (rCBF) in the middle and inferior temporal regions of right cerebral hemisphere in patients with AD. Control subjects showed no significant correlation between thyroid hormone levels and rCBF^46^. The differences in the results from Kimura N. *et al*. with those of our paper could be explained with the differences in the imaging modalities used. Several studies report a superiority of PET as compared to Single Photon Emission Tomography (SPECT) in the diagnosis of AD. In example, O’Brien JT *et al*. reported that sensitivity and specificity for dementia/no-dementia was 85% and 90%, respectively, for 18F-FDG PET and 71% and 70%, respectively, for SPECT^47^. This difference is explained by the higher spatial resolution of PET as compared to SPECT, count sensitivity of detector configurations and by other instrumentation parameters, acquisition and processing techniques, methods and quality of image display^48^. These technical differences could affect the detection of abnormalities of the radiolabeled compounds distributions especially in small mesial structures (BA32 and 25, Table 3) that are detectable by means of PET and not with SPECT. Moreover, despite both imaging techniques proved to be useful in the evaluation of neurodegenerative disorders as AD, the metabolic processes are widely different and to date F-18 FDG is considered as one of the earliest imaging biomarkers of the disease^49^ with changes in brain glucose metabolism being more pronounced than perfusion abnormalities in AD^48^. On the other hand, a possible relationship between the blood flow distribution changes described in the literature and a hypometabolism in the anterior cingulate cortex and left frontal lobe showed in our population should be considered. The demonstration of the hypothesis request an additional research aimed to investigate the relationships between SPECT and PET data in a selected cohort of AD subjects.

As a previous study has shown^50^, also our AD patients displayed an abnormal biofeedback regulation in hypothalamic-pituitary-thyroid axis. Indeed, we found that AD patients showed a trend towards reduced TSH and fT4 levels, and significantly lower fT3 levels compared to controls. Moreover, we found also a loss of correlation between TSH and fT3 or fT4, with regard to the control subjects, further supporting the concept of an abnormal function of the hypothalamic-pituitary-thyroid axis in AD. Interestingly, the decreased glucose metabolism of anterior cingulate cortex and of the medial frontal gyrus correlated positively with variations in serum TSH levels. Several laboratory studies demonstrated a relationship between thyroid state and factors associated with the pathogenesis of AD, including beta-amyloid deposition and neuronal apoptosis^51^. Moreover, thyrotropin releasing hormone (TRH) may affect acetylcholine synthesis and release in the cholinergic neurons of basal forebrain, which are early affected in AD^52^. Furthermore, previous studies^53^ showed a reduction in hypothalamic neuronal populations, with shortened dendrites and dystrophic axons, suggesting that this decreased neural density might contribute not only to TRH release, but also to an alteration in circadian rhythms and instability of autonomic regulation. Experiments in animals have shown that after thyroxine application, the mRNA expression of neurotrophins of the nerve-growth-factor (NGF) family is significantly upregulated both in septum and hippocampus, thus suggesting that modulation of neurotrophin expression contributes to the morphological modifications within the hippocampal mossy fiber system and the septo-hippocampal cholinergic system^54^. From these and recent data, it could be speculated that a reduction of TRH and TSH and other thyroid hormones could affect the synaptic plasticity in AD subjects. Unfortunately, our study lack of a follow up PET/CT examination (i.e. after 1 or 2 years) that could be helpful for a longitudinal assessment of cortical metabolism in relationship to baseline thyroid hormones concentration in our AD population.

The AD-related anterior cingulate cortex involvement was supported also by the negative correlation between its glucose metabolism and CSF t-Tau levels.

Nevertheless, it remains unclear whether altered TSH levels results from or contribute to the development of AD pathology. In this context, the results from our connectivity analysis may support the hypothesis that the abnormal function of the hypothalamic-pituitary-thyroid axis in AD patients reflect a consequence of impairment of the connectivity between medial frontal gyrus and hypothalamus, with a secondary dysfunction of synthesis and release thyrotropin releasing hormone (TRH). Although caution should be taken in attempting to relate these findings without further inquiry, this hypothesis seems to be in accordance with the evidence that a dysfunction of the hypothalamic-pituitary-thyroid axis could be associated to AD and further contribute to cognitive impairment in patients with this neurodegenerative disease.

Certainly, it is important to acknowledge the potential limitations of this study. First, no significant differences in TSH levels were found between AD and CG groups. However, the loss of correlation between TSH and fT3 or fT4 probably reflect an AD-related hypothalamic-pituitary-thyroid axis involvement. Further studies considering also the TRH measurements will be necessary to confirm our results in AD patients. Moreover, some endogenous factors are known to negatively affect TRH release. In particular, among these are the neurotransmitter dopamine, somatostatin and the cytokines IL 1-beta, IL 6 and TNF alpha^55–57^. It has been recently pointed out that neuroinflammation, especially in those subjects with external risk factors as systemic inflammation and obesity, is likely to interfere with immunological processes of the brain and further promote disease progression of AD^58^. More recently, it has been shown that blood brain barrier dysfunction (that has been reported to reflect neuroinflammation in AD^59^) is related to a reduced cortical metabolism in left temporal lobe thus suggesting that a more severe cortical dysfunction occur in those AD subjects with neuroinflammation^60^. Moreover, glucocorticoids and estrogens are known to affect TSH release with stress-related cortisol increase playing a significant role on TSH secretion^57^. Future studies, possibly on a larger cohort of AD subjects, are necessary in order to evaluate the possible relationships of these biomarkers with TRH, TSH fT3 and fT4 levels in AD.

Our study is limited by the cross-sectional design. Future longitudinal studies should confirm our findings, in order to clarify the relationship between TSH and cerebral glucose metabolism in AD patients.

This study demonstrates that the combined use of thyroid hormones detection and PET/CT can help to better explore the biological correlates of hypothalamic-pituitary-thyroid axis dysfunction in AD patients. In particular, our results suggest the involvement of the hypothalamic-pituitary-thyroid axis in the cerebral glucose metabolism changes. Therefore, future investigations considering TRH detection (e.g., internal and external parts) or exploiting also other structural imaging techniques, as magnetic resonance spectroscopy, could add further information to the present findings.

As a last aspect, considering that lower TSH levels were found to be predictive factors of AD progression in subjects with Mild Cognitive Impairment^18^, from a more clinical point of view, the results of our study suggest that TSH levels within the normal range could be considered during the clinical visit and re-visit of the AD patient since this relatively cheap and widely used biomarker could represent a surrogate indicator of neurological estate of the patient and possibly of disease progression.
